# ANP32B, or not to be, that is the question for influenza virus

**DOI:** 10.7554/eLife.48084

**Published:** 2019-06-10

**Authors:** Steven F Baker, Andrew Mehle

**Affiliations:** Department of Medical Microbiology and ImmunologyUniversity of Wisconsin-MadisonMadisonUnited States

**Keywords:** influenza, ANP32A, ANP32B, polymerase, host specificity, evolution, Chicken, Human, Virus

## Abstract

The evolutionary history of ANP32 proteins impacts how influenza virus jumps from birds to mammals.

**Related research article** Long JS, Idoko-Akoh A, Mistry B, Goldhill D, Staller E, Schreyer J, Ross C, Goodbourn S, Shelton H, Skinner MA, Sang H, McGrew MJ, Barclay W. 2019. Species specific differences in use of ANP32 proteins by influenza A virus. *eLife* **8**:e45066. doi: 10.7554/eLife.45066

Every year, the influenza A virus causes flu epidemics around the world, and is also responsible for deadly pandemics like that of 1918. Like most viruses, it must hijack host cells to express its genes, replicate its genome, and produce new virions to infect more cells. Diverse populations of influenza A viruses circulate in birds, their natural reservoir, before emerging in humans and other mammals. As the bird viruses adapt to non-avian species, they face two major barriers: entering the cells of their new hosts, and being able to use their polymerase complex to replicate their genomes (Te Velthuis and Fodor, 2016; Long et al., 2019a). Entry into cells is controlled by receptors that the viruses must bind to, with different species expressing different variants of the receptors. However, the mechanism that blocks the activity of the viral polymerase in a new host, thus preventing the virus from replicating, is less well understood. Looking into the ways that influenza A virus infects new species, and how the hosts fight back, offers a glimpse into complex evolutionary processes in action.

The influenza polymerase is a complex formed of three proteins including one called PB2 (Te Velthuis and Fodor, 2016). Avian-style polymerases work well in bird cells, but poorly in humans. This species-specific restriction is due to the amino acid at position 627 in the PB2 subunit, which is a glutamate in birds and a lysine in humans (Almond, 1977; Subbarao et al., 1993). Changing this glutamate to lysine in an avian polymerase is sufficient to make it active in human cells, but the reasons for this were unclear.

The viral polymerase must recruit proteins from the host in order to transcribe and replicate the viral genome. In 2016, Wendy Barclay of Imperial College and colleagues finally identified ANP32A, the long-sought host co-factor that controls species specificity (Long et al., 2016). There are up to four members of the ANP32 family in vertebrates (A, B, C, and E), all of which have a whip-like shape (Figure 1). Rigorous biochemistry showed that the ANP32 proteins were key co-factors of the viral polymerase in human cells, with ANP32A and ANP32B having redundant roles (Sugiyama et al., 2015). However, just one member of the family in chickens – ANP32A – was able to stimulate avian polymerases; expressing chicken *ANP32A* in human cells rescued activity of the viral polymerases in an otherwise inhospitable species.

**Figure 1.**
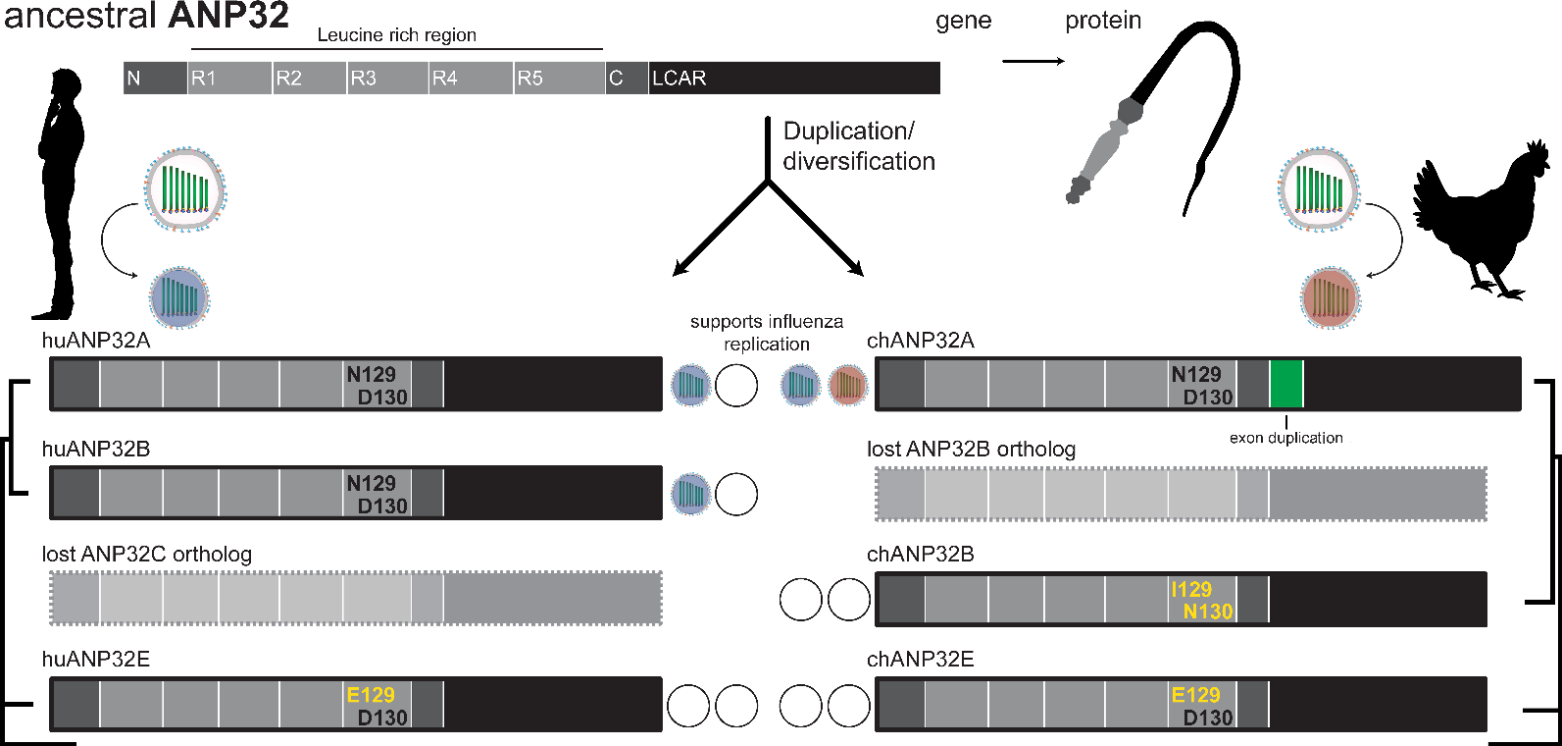
The evolution of ANP32 proteins. ANP32 proteins have a whip-like shape, with an N-terminal region that is rich in leucine (light gray) and a low-complexity acidic region (LCAR; black). Different species bear different ANP32 proteins. The polymerase of the influenza A virus adapts to the ANP32 proteins that are present in different species so that it can replicate in the cells of a new host. Open circles indicate that an ANP32 protein is unable to support viral replication. In humans, ANP32A, 32B and 32E are present, and the influenza virus can use either ANP32A or ANP32B to support replication (blue circles). Human *ANP32C* is a pseudogene. In chicken, ANP32A, 32B and 32E are present, and the influenza virus relies exclusively on ANP32A (red circle). Open circles indicate that an ANP32 protein is unable to support viral replication. An exon duplication in *ANP32A* in chickens (green box) is required for the avian viral polymerase to work. Evolutionary analysis revealed that the avian gene previously identified as *ANP32B* in chickens is most closely related to *ANP32C* genes, as indicated by the trees on the outside of the diagram that show species-specific relationships between *ANP32* genes. Long et al. and Zhang et al. identified the key residues that must be present in the ANP32 proteins for the viral polymerase to be active: asparagine (**N**) at site 129 and aspartate (**D**) at site 130. These amino acids are absent from human ANP32E and from chicken ANP32B and ANP32E.

However, it was not that human cells lacked *ANP32A*; rather, a duplication and insertion in chicken *ANP32A* added a unique exon to the gene. The insertion was common to almost all ANP32A proteins across birds and it was sufficient to confer activity to polymerases of avian origin. Subsequent studies began to dissect the activity of ANP32A and identified key features of this insert. In particular, they showed that, in birds, *ANP32A* is differentially spliced at the insert, creating ANP32A variants with different abilities to support polymerase function (Baker et al., 2018; Domingues and Hale, 2017). Thus, multiple lines of evidence converged on the importance of ANP32 proteins and their potential role in the evolution of influenza viruses. Now, in eLife, Barclay and colleagues at Imperial College, Edinburgh University, St George's in London and the Pirbright Institute – including Jason Long as first author – report, alongside a paper by Xiaojun Wang and colleagues in the Journal of Virology, that ANP32 proteins may have an even more intriguing evolutionary history (Long et al., 2019b; Zhang et al., 2019).

First, the experiments confirmed that ANP32A and ANP32B have a redundant role in helping the influenza virus to replicate in human cells. The double-knockout of *ANP32A* and *ANP32B* almost completely eliminated polymerase activity and virus replication, but simply deleting ANP32A had no effect (Long et al., 2019b; Zhang et al., 2019). HoweverIn contrast, removing only ANP32A in chickens was sufficient to stop the viral polymerase from working (Long et al., 2019b). Taken together, these results imply that both ANP32A and ANP32B support infection in humans, but only ANP32A performs this role in chickens, with ANP32B being unable to help the polymerase. Finally, neither human nor chicken ANP32Es played a major role during viral replication.

An in-depth investigation of ANP32B in chicken revealed that everything was not as it seemed. Evolutionary analyses showed that avian *ANP32B* forms a distinct group, and may actually be more closely related to *ANP32C* genes (and perhaps should be referred to as such) (Figure 1; Long et al., 2019b). Birds lost or never possessed the *ANP32B* version found in mammals, while the *ANP32C* gene that is closely related to avian *ANP32B* emerged in placental mammals as a non-functional pseudogene. Both Long et al. and Zhang et al. exploited their knockout cells to show that only two amino acid changes in chicken ANP32B eliminate its ability to support the viral polymerase (Figure 1). Avian ANP32A has asparagine and aspartate at residues 129 and 130, whereas avian ANP32B has isoleucine and asparagine at the same sites. These differences control the ability of the protein to interact with and promote the activity of the viral polymerase. Human ANP32A and ANP32B both have asparagine and aspartate at these sites, explaining why either protein supports the activity of the viral polymerase. These studies provide compelling evidence that subtle changes in ANP32 proteins can have large impacts on how viruses can exploit these proteins to replicate.

It is still unclear which evolutionary forces drove the duplication and subsequent diversification of the *ANP32* loci, while also fixing the insertion in chicken *ANP32A*. How ANP32 proteins support the activity of the influenza polymerase also remains to be elucidated. In addition, it is likely that selective pressures on ANP32 proteins were not unique to birds. Mouse ANP32A, for example, only marginally supports polymerase activity (Zhang et al., 2019). In this species, ANP32A has asparagine and alanine at residues 129 and 130, a variation that the current studies would predict to be non-functional for the influenza virus. Instead, the mouse variant of ANP32B that has serine and aspartic acid at these sites is likely the dominant form which supports infection. It is curious that similar changes in ANP32A arise in pangolins, a species whose evolution is very distant from rodents and which has no obvious history with influenza virus. "For who would bear the whips of scorn and time", cries out Hamlet in the eponymous play. And indeed, the whip-like ANP32 proteins bear the telltale signs of mutation and diversification that suggest a long history of viruses exploiting these proteins.

## References

[bib1] Almond JW (1977). A single gene determines the host range of influenza virus. Nature.

[bib2] Baker SF, Ledwith MP, Mehle A (2018). Differential splicing of ANP32A in birds alters its ability to stimulate RNA synthesis by restricted influenza polymerase. Cell Reports.

[bib3] Domingues P, Hale BG (2017). Functional insights into ANP32A-dependent influenza A virus polymerase host restriction. Cell Reports.

[bib4] Long JS, Giotis ES, Moncorgé O, Frise R, Mistry B, James J, Morisson M, Iqbal M, Vignal A, Skinner MA, Barclay WS (2016). Species difference in ANP32A underlies influenza A virus polymerase host restriction. Nature.

[bib5] Long JS, Mistry B, Haslam SM, Barclay WS (2019a). Host and viral determinants of influenza A virus species specificity. Nature Reviews Microbiology.

[bib6] Long JS, Idoko-Akoh A, Mistry B, Goldhill D, Staller E, Schreyer J, Ross C, Goodbourn S, Shelton H, Skinner MA, Sang H, McGrew MJ, Barclay W (2019b). Species specific differences in use of ANP32 proteins by influenza A virus. eLife.

[bib7] Subbarao EK, London W, Murphy BR (1993). A single amino acid in the PB2 gene of influenza A virus is a determinant of host range. Journal of Virology.

[bib8] Sugiyama K, Kawaguchi A, Okuwaki M, Nagata K (2015). pp32 and APRIL are host cell-derived regulators of influenza virus RNA synthesis from cRNA. eLife.

[bib9] Te Velthuis AJ, Fodor E (2016). Influenza virus RNA polymerase: insights into the mechanisms of viral RNA synthesis. Nature Reviews Microbiology.

[bib10] Zhang H, Zhang Z, Wang Y, Wang M, Wang X, Zhang X, Ji S, Du C, Chen H, Wang X (2019). Fundamental contribution and host range determination of ANP32A and ANP32B in influenza A virus polymerase activity. Journal of Virology.

